# 

**Published:** 1890-05

**Authors:** 


### CONTENTS—MAY, 1890.—VOL. 5, NO. 11.

Twenty-second Annual Session of the Texas State Medical Association—Synopsis of Proceedings . . . . . . . . . . 407
New Members . . . . . . . . . . . . . . . . . . . . . . 431

ORIGINAL COMMUNICATIONS—

Complete Closure of Both External Auditory Canals by Bone. J. W. Carhart, M. D. . . . . . . . . . . . . . . . 432

CORRESPONDENCE—

Letter from Dr. Leake . . . . . . . . . . . . . . . . . 434

SOCIETY NOTES—

Taylor County Medical Society . . . . . . . . . . . . . 437

EDITORIAL—

The Recent Meeting of the Texas State Medical Association 439
Notes of the Convention . . . . . . . . . . . . . . . . 441
Hastening Slowly . . . . . . . . . . . . . . . . . . . . 442
Two Committees on Medical Legislation . . . . . . . . . 444

MEDICAL NEWS AND MISCELLANY—

Various Items of Interest . . . . . . . . . . . . . . . 444
PUBLISHER'S NOTES . . . . . . . . . . . . . . . . . . . 447

